# Sibling Death in Childhood and Early Adulthood and Risk of Early-Onset Cardiovascular Disease

**DOI:** 10.1001/jamanetworkopen.2023.50814

**Published:** 2024-01-08

**Authors:** Chen Huang, Jiahuan Peng, Priscilla Ming Yi Lee, Ce Wang, Kecheng Wei, Minhong Liang, Guoyou Qin, Yongfu Yu, Jiong Li

**Affiliations:** 1 Department of Biostatistics, Key Laboratory for Health Technology Assessment, National Commission of Health, Key Laboratory of Public Health Safety of Ministry of Education, School of Public Health, Fudan University, Shanghai, China; 2 JC School of Public Health and Primary Care, the Chinese University of Hong Kong, Hong Kong Special Administrative Region, China; 3 Shanghai Hongkou Center for Disease Control and Prevention, Shanghai, China; 4 Shanghai Institute of Infectious Disease and Biosecurity, Shanghai, China; 5 Department of Epidemiology, School of Public Health, Nanjing Medical University, Nanjing, China

## Abstract

**Importance:**

Sibling death is a highly traumatic event, but empirical evidence on the association of sibling death in childhood and early adulthood with subsequent risk of incident cardiovascular disease (CVD) remains limited.

**Objective:**

To evaluate the association between sibling death in the early decades of life and subsequent risk of incident early-onset CVD.

**Design, Setting, and Participants:**

This population-based cohort study included 2 098 659 individuals born in Denmark from 1978 to 2018. Follow-up started at age 1 year or the date of the first sibling’s birth, whichever occurred later, and it ended at the first diagnosis of CVD, the date of death, emigration, or December 31, 2018, whichever came first. Data analyses were conducted from November 1, 2021, through January 10, 2022.

**Exposures:**

The death of a sibling.

**Main Outcomes and Measures:**

The outcome was early-onset CVD. Cox models were used to estimate hazard ratios (HRs) with 95% CIs.

**Results:**

This study included 2 098 659 individuals (1 076 669 [51.30%] male; median [IQR] age at death of sibling, 11.48 [4.68-21.32] years). During the median (IQR) follow-up of 17.52 (8.85-26.05) years, 1286 and 76 862 individuals in the bereaved and nonbereaved groups, respectively, were diagnosed with CVD. Sibling death in childhood and early adulthood was associated with a 17% increased risk of overall CVD (HR, 1.17; 95% CI, 1.10-1.23; cumulative incidence in bereaved individuals, 1.96% [1.61%-2.34%]; cumulative incidence in nonbereaved individuals at age 41 years, 1.35% [1.34%-1.37%]; cumulative incidence difference: 0.61% [95% CI, 0.24%-0.98%]). Increased risks were also observed for most type-specific CVDs, in particular for myocardial infarction (HR, 1.66; 95% CI, 1.12-2.46), ischemic heart disease (HR, 1.52; 95% CI, 1.22-1.90), and heart failure (HR, 1.50; 95% CI, 1.00-2.26). The association was observed whether the sibling died due to CVD (HR, 2.54; 95% CI, 2.04-3.17) or non-CVD (HR, 1.13; 95% CI, 1.06-1.19) causes. The increased risk of CVD was more pronounced for individuals who lost a twin or younger sibling (HR, 1.25; 95% CI, 1.15-1.36) than an elder sibling (HR, 1.11; 95% CI, 1.03-1.20).

**Conclusions and Relevance:**

In this cohort study of the Danish population, sibling death in childhood and early adulthood was associated with increased risks of overall and most type-specific early-onset CVDs, with the strength of associations varying by cause of death and age difference between sibling pairs. The findings highlight the need for extra attention and support to the bereaved siblings to reduce CVD risk later in life.

## Key Points

**Question:**

Is there an association of sibling death in childhood and early adulthood with subsequent risk of incident cardiovascular disease (CVD)?

**Findings:**

In this cohort study of more than 2.0 million participants born in Denmark, sibling death in childhood and early adulthood was associated with increased risks of overall and most specific early-onset CVDs among bereaved siblings, with strength of associations varying by cause of death and age difference between sibling pairs.

**Meaning:**

These findings suggest that individuals who experience the death of a sibling in childhood may have greater risk of early-onset CVD; extra support to these individuals may help mitigate the potential adverse effects of bereavement on cardiovascular health.

## Teaser

This cohort study evaluates the association between sibling death in the early decades of life and subsequent risk of incident early-onset cardiovascular disease (CVD) in the Danish population.

## Introduction

The incidence and prevalence of CVD have been increasing over the past few decades among children, adolescents, and young adults,^1,2^ which poses a concerning public health burden and economic loss.^2,3,4,5^ Previous studies have suggested that psychological stress following bereavement may contribute to the development of CVD.^6,7^ The loss of a spouse, a child, a parent, or other family members was associated with increased risks of type-specific CVDs, including ischemic heart disease (IHD), stroke, atrial fibrillation (AF), myocardial infarction (MI), pulmonary embolism, cerebrovascular disease, and heart failure.^8,9,10,11,12,13,14,15,16,17,18,19^

The sibling relationship is among the longest and most intimate in life, exerting a unique and profound influence on each other’s development during childhood and adolescence, alongside other important relationships.^20,21^ Previous studies have suggested that the loss of a sibling is a highly traumatic event and may be more disruptive than the loss of other family members.^21,22^ However, the surviving siblings frequently received inadequate attention, and to our knowledge, there is no existing evidence regarding the impact of sibling death in childhood and early adulthood on the surviving sibling’s subsequent risk of overall and specific CVDs, except 2 studies from Sweden^23,24^ that reported an association between sibling death in adulthood and an increased risk of mortality from fatal stroke or MI. Additionally, sibling contact frequency and closeness may vary across life stages, which may be influenced by age gap between siblings.^25,26^ Furthermore, grief experienced by children and adolescents following bereavement may evolve over time with varying patterns.^27^ Moreover, the association between sibling death and CVD incidence may be affected by shared genetic, childhood environmental, and living factors.^28,29,30,31,32^ Therefore, examining sibling deaths due to specific causes may offer a deeper understanding of the potential link between sibling death and subsequent risk of early-onset CVD.

In this large population-based study in Demark, we investigated the association between the death of a sibling in the early decades of life and subsequent risk of overall and specific CVDs. We further examined whether the association depended on the cause of sibling death, age difference between sibling pairs, time since sibling death, and age at bereavement.

## Methods

### Study Population

We conducted a population-based cohort study that included all individuals born in Denmark from 1978 to 2018 (N = 2 418 760), using data from several national registers (eAppendix 1 in Supplement 1).^33,34^ Foreign-born individuals were not included. The flowchart of study participants appears in eFigure 1 in Supplement 1. We linked individuals with their mothers to identify their siblings, and 2 209 187 individuals had at least 1 sibling. Considering that CVD occurring before age 1 year may often be attributable to maternal or prenatal conditions other than sibling death,^29,35^ we excluded individuals born after December 31, 2017, or the sibling’s death (n = 43 637) as well as individuals who died (n = 14 035) or emigrated (n = 2833) before age 1 year or the first sibling’s birth. Individuals diagnosed with CVD at or before the date of sibling’s death, at 1 year, or the first sibling’s birth were also excluded (n = 8161). We further excluded individuals diagnosed with congenital heart disease (n = 36 236) or with unknown sex (n = 5626). The final analysis included 2 098 659 study participants. Follow-up started at age 1 year or the date of the first sibling’s birth, whichever occurred later, and it ended at the first diagnosis of CVD, the date of death, emigration, or December 31, 2018, whichever came first. The study was approved by the Data Protection Agency. By Danish law, no informed consent is required for a register-based study based on anonymized data. The study followed the Strengthening the Reporting of Observational Studies in Epidemiology (STROBE) reporting guideline.

### Exposure

We defined exposure as the death of a sibling occurring after the start of follow-up. The exposure was treated as a time-varying variable: all individuals were included in the unexposed group at first and would be changed to the exposed group when the individual lost a sibling. If multiple siblings died at different times, exposure time started at the death date of the first deceased sibling.

The cause of sibling death was retrieved from the Danish Cause of Death Register using the *International Classification of Diseases* codes (*International Classification of Diseases, Eighth Revision *[*ICD-8*], used 1978-1993, codes 000-E999; *International Statistical Classification of Diseases and Related Health Problems, Tenth Revision *[*ICD-10*], used 1994 and forward, codes A00-Y89).^34^ Considering that the association between sibling death and CVD may be influenced by shared biological and genetic similarities among sibling pairs,^28,29,30,31,32^ we further categorized the cause of the death into death due to CVD (*ICD-8* codes, 390–458; *ICD-10* codes, I00–I99) or death due to non-CVD causes.

### Outcome of Interest

The outcome of interest was incident early-onset CVD, defined as the first occurrence of CVD in the Danish National Patient Register (DNPR) and the Danish Cause of Death Register in childhood and early adulthood (age 0 to 41 years).^34^ We further investigated type-specific CVDs, including IHD, MI, cerebrovascular disease, stroke, ischemic stroke, heart failure, AF, hypertensive disease, deep vein thrombosis, pulmonary embolism, rheumatic heart disease, and peripheral artery disease (specific *ICD* codes are provided in eTable 1 in Supplement 1).

### Covariates

Potential confounders were selected based on directed acyclic graph (eFigure 2 in Supplement 1), including preterm birth (<37 weeks, ≥37 weeks, or unknown), birth weight (<2500 g, 2500-3249 g, 3250-3999 g, ≥4000 g, or unknown), singleton birth (yes or no), parity (1, 2, or ≥3), Apgar score at 5 minutes (0-6, 7-9, 10, or unknown), maternal age (<20 years, 20-24 years, 25-29 years, 30-34 years, or ≥35 years), maternal education (0-9 years, 10-14 years, ≥15 years, or unknown), maternal smoking during pregnancy (yes or no), sex of child (male or female), and calendar period (1980 and before, a 10-year interval during 1980-2009, 2010 and after), maternal hypertensive disorder of pregnancy (yes or no), parental history of CVD (yes or no), and parental history of diabetes (yes or no) before child birth. Data on birth weight and maternal education were available during 1979 to 2018; maternal smoking was available during 1991 to 2018; other covariates were available during 1978 to 2018. Multiple imputation with 10 replications was used to deal with missing values.^36^ Detailed descriptions of covariates, missing data, and multiple imputation are presented in eAppendix 2 and eTables 2 and 3 in Supplement 1.

### Statistical Analysis

Considering non-CVD deaths as competing events, we estimated cumulative incidence of overall CVD among individuals exposed and unexposed to sibling death. Cox regression, with sibling death as a time-dependent variable, was used to estimate hazard ratios (HRs) with 95% CIs. Robust variance was used to account for the correlations between surviving siblings in the same familty.^37^ The proportional hazard assumption was not violated according to the log-minus-log plot (eFigure 3 in Supplement 1).

We performed analyses with exposure categorized according to the type of sibling death (death due to CVD or non-CVD cause) and age difference between sibling pairs (death of an elder sibling or death of a twin or younger sibling, which were further categorized into age difference of 0-6 years or >6 years). We also performed analyses jointly stratified by age at bereavement (<6 years, 6-11 years, 12-17 years, or ≥18 years, to account for the different developmental stages of young childhood, childhood, adolescence, and young adulthood) and time since bereavement (<1 years, 1-4 years, 5-9 years, 10-14 years, or ≥15 years).

We performed several sensitivity analyses to test the robustness of our results. First, we performed analyses of associations between sibling death and type-specific CVDs stratified on cause of sibling death. Second, we performed mediation analysis to examine how mental disorders might mediate the association of sibling death with CVD. Third, we conducted analyses stratified by sex of the bereaved person. Fourth, we performed analyses restricted to full siblings (1 902 122 participants), which were defined as siblings from both the same mother and father;. Finally, we also undertook several subgroup analyses: additionally adjusting for parental mental disorders; adjusting for minimal adjustment sets (calendar year, maternal hypotensive disorder of pregnancy, maternal education, maternal smoking, parental CVD, and parental diabetes); follow-up beginning at birth, age 6 months, age 3 years, and age 5 years; analyses restricted to individuals born after 1980, 1991, and 1994 to consider the influence of data availability of covariates and *ICD* code changes (*ICD-10* was adopted in 1994); complete cases analysis; using restricted cubic splines to fit the potential nonlinear association between continuous covariates (birth weight, Apgar score, maternal age, and calendar year) and CVD risk. All analyses were performed using SAS version 9.4 (SAS Institute) and Stata version 14 (StataCorp). A 2-sided *P* < .05 determined statistical significance. Data analyses were conducted from November 1, 2021, through January 10, 2022.

## Results

Among 2 098 659 study participants (1 076 669 [51.30%] males; median [IQR] age at sibling death, 11.48 [4.68-21.32] years), 22 968 (1.09%) lost a sibling during follow-up. The maximum age for bereavement was 40.85 years. Bereaved siblings were more likely to have a mother who was younger at pregnancy, had lower education level, had higher parity, and smoked during pregnancy. Bereaved individuals were more likely to be born preterm and have a lower birth weight (Table 1).

**Table 1.** Baseline Characteristics of the Study Population

Characteristic	Study cohort, No. (%)		
	Exposed to sibling death (n = 22 968)	Unexposed to sibling death (n = 2 075 691)	Total (N = 2 098 659)
Sex			
Male	11 751 (51.2)	1 064 918 (51.3)	1 076 669 (51.3)
Female	11 217 (48.8)	1 010 773 (48.7)	1 021 990 (48.7)
Preterm birth			
No	20 022 (87.2)	1 896 340 (91.4)	1 916 362 (91.3)
Yes	1573 (6.8)	111 775 (5.4)	113 348 (5.4)
Unknown	1373 (6.0)	67 576 (3.3)	68 949 (3.3)
Birth weight, g^a^			
<2500	1491 (6.9)	95 877 (4.7)	97 368 (4.7)
2500-3249	6685 (30.8)	521 699 (25.7)	528 384 (25.8)
3250-3999	10 200 (46.9)	1034 624 (51.0)	1 044 824 (50.9)
≥4000	3181 (14.6)	352 882 (17.4)	356 063 (17.4)
Unknown	182 (0.8)	24 007 (1.2)	24 189 (1.2)
Singleton			
No	679 (3.0)	76 128 (3.7)	76 807 (3.7)
Yes	22 289 (97.0)	1 999 563 (96.3)	2 021 852 (96.3)
Parity			
1	8985 (39.1)	821 536 (39.6)	830 521 (39.6)
2	7266 (31.6)	855 718 (41.2)	862 984 (41.1)
≥3	6717 (29.2)	398 437 (19.2)	405 154 (19.3)
Apgar score at 5 min			
0-6	168 (0.7)	13 994 (0.7)	14 162 (0.7)
7-9	1340 (5.8)	122 001 (5.9)	123 341 (5.9)
10	20 928 (91.1)	1 899 260 (91.5)	1 920 188 (91.5)
Unknown	532 (2.3)	40 436 (1.9)	40 968 (2.0)
Maternal age at birth, y			
15-19	1304 (5.7)	46 359 (2.2)	47 663 (2.3)
20-24	6280 (27.3)	362 676 (17.5)	368 956 (17.6)
25-29	8065 (35.1)	766 762 (36.9)	774 827 (36.9)
30-34	5066 (22.1)	629 473 (30.3)	634 539 (30.2)
≥35	2253 (9.8)	270 421 (13.0)	272 674 (13.0)
Maternal education at birth, y^a^			
0-9	10 065 (46.3)	518 861 (25.6)	528 926 (25.8)
10-14	7649 (35.2)	882 853 (43.5)	890 502 (43.4)
≥15	3606 (16.6)	604 830 (29.8)	608 436 (29.7)
Unknown	419 (1.9)	22 545 (1.1)	22 964 (1.1)
Maternal smoking during pregnancy^b^			
No	5810 (62.7)	1 137 720 (77.7)	1 143 530 (77.6)
Yes	2880 (31.1)	265 235 (18.1)	268 115 (18.2)
Unknown	578 (6.2)	60 915 (4.2)	61 493 (4.2)
Parents with CVD history			
No	22 251 (96.9)	1 969 194 (94.9)	1 991 445 (94.9)
Yes	717 (3.1)	106 497 (5.1)	107 214 (5.1)
Maternal gestational hypertension			
No	21 808 (94.9)	1 962 207 (94.5)	1 984 015 (94.5)
Yes	1160 (5.1)	113 484 (5.5)	114 644 (5.5)
Parents diabetes history			
No	22 694 (98.8)	2 040 158 (98.3)	2 062 852 (98.3)
Yes	274 (1.2)	35 533 (1.7)	35 807 (1.7)

Abbreviation: CVD, cardiovascular disease.

^a^ Birth weight and maternal educational level data were available in Denmark from 1979 to 2018; therefore, the percentage was calculated based on participants born after 1979 (n = 2 050 828).

^b^ Maternal smoking data during pregnancy was available in Denmark from 1991 to 2018; therefore the percentage was calculated based on participants born after 1991 (n = 1 473 138).

**Figure 1.**
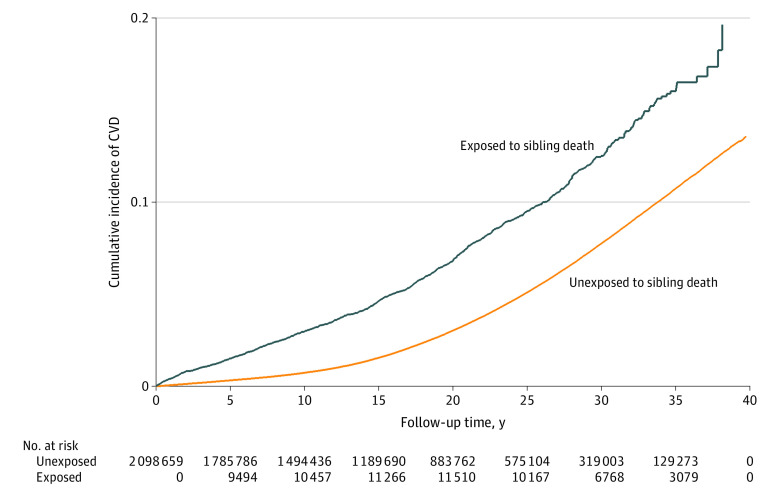
Cumulative Incidence of Cardiovascular Disease (CVD) Among Individuals With and Without the Death of a Sibling

During the median (IQR) follow-up period of 17.52 (8.85-26.05) years, 1286 and 76 862 individuals were diagnosed with CVD in the bereaved and nonbereaved groups, respectively (Figure 1 and Table 2). The median (IQR) age of CVD onset was 22.86 (16.65-28.91) years. Bereaved individuals had a 17% higher risk of overall CVD than nonbereaved individuals (HR, 1.17; 95% CI, 1.10-1.23; cumulative incidence in nonbereaved individuals at age 41 years: 1.35% [95% CI, 1.34%-1.37%]; cumulative incidence in bereaved individuals, 1.96% [1.61%-2.34%]; cumulative incidence difference: 0.61% [95% CI, 0.24%-0.98%]). The risks of most type-specific CVDs were also increased, especially for MI (HR, 1.66; 95% CI, 1.12-2.46), IHD (HR, 1.52; 95% CI, 1.22-1.90) and heart failure (HR, 1.50; 95% CI, 1.00-2.26) (Table 2).

**Table 2.** Associations Between Sibling Death and Overall CVD and Type-Specific CVDs

Outcome and exposure	Observations, No.	CVD cases, No.	Rate per 1000 person-years	cHR (95% CI)	aHR (95% CI)^a^
**Overall CVD**					
Unexposed	2 075 691	76 862	2.08	1 [Reference]	1 [Reference]
Exposed	22 968	1286	3.86	1.19 (1.13-1.26)	1.17 (1.10-1.23)
**Ischemic heart disease**					
Unexposed	2 080 860	2681	0.07	1 [Reference]	1 [Reference]
Exposed	23 445	80	0.23	1.70 (1.36-2.13)	1.52 (1.22-1.90)
**Myocardial infarction**					
Unexposed	2 080 887	698	0.02	1 [Reference]	1 [Reference]
Exposed	23 455	26	0.08	1.88 (1.27-2.78)	1.66 (1.12-2.46)
**Cerebrovascular disease**					
Unexposed	2 080 699	2817	0.07	1 [Reference]	1 [Reference]
Exposed	23 439	61	0.18	1.43 (1.11-1.84)	1.34 (1.04-1.73)
**Stroke**					
Unexposed	2 080 655	1060	0.03	1 [Reference]	1 [Reference]
Exposed	23 451	21	0.06	1.60 (1.04-2.47)	1.48 (0.96-2.28)
**Ischemic stroke**					
Unexposed	2 080 640	1861	0.05	1 [Reference]	1 [Reference]
Exposed	23 443	40	0.12	1.32 (0.96-1.80)	1.23 (0.90-1.69)
**Heart failure**					
Unexposed	2 080 806	890	0.02	1 [Reference]	1 [Reference]
Exposed	23 442	24	0.07	1.62 (1.08-2.43)	1.50 (1.00-2.26)
**Atrial fibrillation**					
Unexposed	2 080 862	2066	0.05	1 [Reference]	1 [Reference]
Exposed	23 448	33	0.10	0.94 (0.67-1.33)	0.92 (0.65-1.29)
**Hypertensive disease**					
Unexposed	2 080 698	8623	0.23	1 [Reference]	1 [Reference]
Exposed	23 408	180	0.52	1.24 (1.07-1.43)	1.15 (0.99-1.33)
**Deep vein thrombosis**					
Unexposed	2 080 882	4383	0.12	1 [Reference]	1 [Reference]
Exposed	23 422	107	0.31	1.46 (1.20-1.77)	1.38 (1.14-1.67)
**Pulmonary embolism**					
Unexposed	2 080 847	2169	0.06	1 [Reference]	1 [Reference]
Exposed	23 441	56	0.16	1.55 (1.19-2.02)	1.47 (1.13-1.92)
**Rheumatic heart disease**					
Unexposed	2 080 867	209	0.01	1 [Reference]	1 [Reference]
Exposed^b^	23 456	<6	0.01	1.61 (0.60-4.34)	1.51 (0.56-4.09)
**Peripheral artery disease**					
Unexposed	2 080 887	437	0.01	1 [Reference]	1 [Reference]
Exposed	23 456	6	0.02	0.89 (0.40-1.99)	0.86 (0.38-1.92)

Abbreviations: aHR, adjusted hazard ratio; cHR, crude hazard ratio; CVD, cardiovascular disease.

^a^ Adjusted for preterm birth, birth weight, singleton birth, parity, Apgar score at 5 minutes, maternal age, maternal education, maternal smoking during pregnancy, sex of child, calendar period, maternal hypertensive disorder of pregnancy, parental history of CVD before child birth, and parental history of diabetes before child birth.

^b^ Fewer than 6 cases are not allowed to be reported due to data protection in Denmark.

The risk of CVD was higher if the deceased sibling died due to CVD causes (HR, 2.54; 95% CI, 2.04-3.17), and the increased risks were also observed if the deceased sibling died due to non-CVD causes (HR, 1.13; 95% CI, 1.06-1.19). In terms of the age difference between sibling pairs, the strength of association was stronger for individuals who lost a twin or a younger sibling (HR, 1.25; 95% CI, 1.15-1.36) compared with those who lost an elder sibling (HR, 1.11; 95% CI, 1.03-1.20) (Table 3).

**Table 3.** Associations Between Sibling Death and CVD by Cause of Sibling’s Death and Age Difference Between Sibling Pairs

Exposure	Observations, No.	CVD cases, No.	Rate per 1000 person-years	cHR (95%CI)	aHR (95%CI)^a^
Unexposed	2 075 691	76 862	2.08	1 [Reference]	1 [Reference]
Cause of sibling’s death					
CVD	868	79	10.47	2.62 (2.10-3.26)	2.54 (2.04-3.17)
non-CVD cause	22 100	1207	3.70	1.15 (1.09-1.22)	1.13 (1.06-1.19)
Age difference between sibling pairs					
Elder sibling death					
Any age difference	12 803	733	3.41	1.14 (1.06-1.22)	1.11 (1.03-1.20)
0-6 y	9352	523	3.21	1.13 (1.04-1.23)	1.12 (1.02-1.22)
>6 y	3451	210	4.04	1.15 (1.01-1.32)	1.10 (0.96-1.26)
Twin or younger sibling death					
Any age difference	10 165	553	4.67	1.28 (1.18-1.39)	1.25 (1.15-1.36)
0-6 y	6143	367	4.99	1.27 (1.15-1.41)	1.24 (1.12-1.37)
>6 y	4022	186	4.14	1.29 (1.12-1.49)	1.27 (1.10-1.46)

Abbreviations: aHR, adjusted hazard ratio; cHR, crude hazard ratio; CVD, cardiovascular disease.

^a^ Adjusted for preterm birth, birth weight, singleton birth, parity, Apgar score at 5 minutes, maternal age, maternal education, maternal smoking during pregnancy, sex of child, calendar period, maternal hypertensive disorder of pregnancy, parental history of CVD before child birth, and parental history of diabetes before child birth.

**Figure 2.**
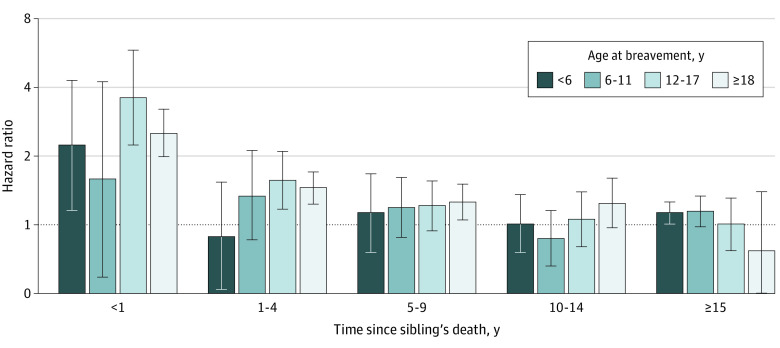
Cardiovascular Disease Hazard Ratios After Sibling Death by Age at Bereavement and Years Since Bereavement

With respect to elapsed time since bereavement, the increased CVD risk after sibling death was observed in the short and long term, regardless of age at bereavement. However, the magnitude of the association seemed to be stronger in the first year after bereavement, especially for those who experienced sibling death in adolescence (HR, 3.60; 95% CI, 2.23-5.80) (Figure 2; eTable 4 in Supplement 1).

The risk of most type-specific CVDs was also higher if the deceased sibling died due to CVD (eTable 5 in Supplement 1). Mediation analysis found that the proportion of the association between sibling death and overall CVD explained by subsequent mental disorder was 21.9% (eTable 6 in Supplement 1). Analysis stratified by sex yielded similar associations (eTable 7 in Supplement 1). Results from subgroup analyses including full-sibling analysis; additionally adjusting for parental mental disorders; adjusting for minimal adjustment sets; different start dates of the follow-up (birth, age 6 months, age 3 years, or age 5 years); restriction to individuals born after 1980, 1991, and 1994; restriction to individuals with complete data; and the analysis using restricted cubic splines for continuous covariates were similar to those of the main analysis (eTable 8-9 in Supplement 1).

## Discussion

In this large population-based cohort study, we found that sibling death in childhood or early adulthood was associated with increased risks of overall or most type-specific CVDs. The association was persistent not only for sibling loss due to CVD but also for non-CVD causes. Stronger associations were found among participants who lost a twin or younger sibling. The increased risk was observed both in the short and long term, with the highest risk observed within the first year after bereavement, especially for those experiencing the death of a sibling in adolescence.

To our knowledge, this is the first population-based study to offer comprehensive insights into the association between sibling death and risk of overall and type-specific CVD incidence during childhood, adolescence, and young adulthood. Prior studies have predominantly focused on sibling death and its subsequent association with mortality, including all-cause mortality, suicide, and mortality from fatal stroke and MI.^23,24,28,29,30,31,32^ Among them, 2 Swedish studies including participants aged 40 to 69 years during 1981 to 2002 found that the loss of an adult sibling was associated with 11% to 31% increased risks of mortality from fatal stroke and MI.^23,24^ We also observed increased risks of MI and stroke, although the increased risk of stroke was not statistically significant. The varying results may be due to the differences in the study period and age distribution of the study population (our study focused on relatively young individuals) as well as difference in the mechanisms of CVD incidence and mortality.^23,24^ In line with previous studies that examined the impact of bereavement other than sibling loss, we observed 34% to 66% higher risks of heart failure, MI, IHD, pulmonary embolism, and cerebrovascular disease among the bereaved who lost a sibling.^8,9,10,11,12,13,14,15,16,17,18^ These findings underscored the profound impact of bereavement on cardiovascular health, and our findings of sibling death and risk of overall CVD and a wider range of cardiovascular events extended the limited literature on bereavement and CVD risk. The differences in the association between sibling death and different type-specific CVDs could stem from different pathobiological mechanisms, which warrant further research.

Considering different causes of sibling death, we found that individuals experiencing sibling loss due to CVD had a higher CVD risk, which supports the hypothesis that common genetic and environmental cardiovascular risk factors that clustered in families are likely to be an important explanation for the association between bereavement and CVD.^9,10,11,12,29,30,31^ Nevertheless, we also observed an increased risk of CVD if the sibling died of a non-CVD cause, which suggests an independent role of stress-related mechanisms between sibling death and CVD incidence.^9,10,11,12,29,30,31^ Psychological stress following bereavement could activate acute stress responses, which may lead to changes in brain stress-responsive neurocircuitry and then stimulate peripheral physiological responses, including the autonomic nervous system response, hypothalamic-pituitary-adrenal responses, and sterile inflammation.^38,39,40^ These stress responses would lead to pathophysiological changes (such as cardiac electrical instability, myocardial ischemia, atherosclerosis, and thrombus formation) over time, contributing to the development and progression of CVD.^39^

Our study observed a stronger association among participants who lost a twin or younger sibling than those who lost an elder sibling. The loss of a younger sibling goes against the common expectation that older individuals tend to die before younger ones.^17,41^ Reports also indicate that the eldest child in a family often assumes greater responsibility for younger siblings, potentially putting them at a disadvantage in emotional adjustment.^42^ In addition, twinship is often considered the closest relationship due to a higher degree of relatedness.^31^ Surviving elder or twin siblings may experience more intense grief, potentially leading to unusual psychophysiological states and abnormal cardiovascular health outcomes.^31,43^

The increased risks persisted over the follow-up period, with the highest risk of CVD occurring within the first year after bereavement. Our finding was consistent with those previous studies that the risks of AF, acute MI, hemorrhagic stroke, and heart failure were the highest immediately after the death of a child or a partner.^8,9,10,11,15,23,24^ The findings of a higher CVD risk shortly after sibling death may support the acute physiological mechanisms for posttraumatic stress on CVD, which included the activation of hypothalamic-pituitary-adrenal axis and short-term increased inflammatory activity.^38,44^ Additionally, our findings might support the roles of short-term lifestyle and behavior changes in the postbereavement period.^45^ In contrast, a Swedish study of individuals born in 1932 to 1962 found a stronger association between sibling death and mortality in the longer term than in the short term,^30^ and a Danish study from 1980 to 1996 only observed an increased risk of MI after 6 years of follow-up in parents who lost a child.^13^ The differing results may be attributed to variations in mechanisms of mortality and CVD incidence, types of bereavement, and study periods.^13,30^ Notably, we observed a more than 3-fold increased risk of CVD within the first year following sibling death during adolescence. Adolescents may have strong emotional bonds with their deceased siblings, and this sensitive period may render them particularly vulnerable to psychosocial adversity. The stress and intense grief associated with bereavement may trigger CVD events shortly after the loss.^28,46^ We also observed that the risk of CVD remained elevated 5 to 10 years after sibling death. This suggests that sibling death may result in prolonged grief and stress among the surviving siblings, leading to chronic adverse effects on cardiovascular health.^25,47^

### Strengths and Limitations

This study had several notable strengths. First, it was conducted based on high-quality data from Danish national registers, which minimized the possibility of recall bias or selection bias.^34^ Second, the large sample size and extended follow-up of up to 41 years enabled us to explore the association between sibling death and 12 specific types of CVD as well as to assess the potential variations based on age at bereavement and time since exposure. Third, we analyzed various causes of sibling death to account for potential genetic and unmeasured familial factors.

Several limitations need to be acknowledged. First, while we adjusted for numerous key confounding factors, the potential for residual confounding by unmeasured variables, including environmental and genetic factors among sibling pairs, cannot be entirely ruled out. However, the association between a sibling’s non-CVD death and CVD incidence suggests that genetic factors alone are unlikely to completely explain the association of sibling death with CVD. Second, CVD diagnosis from primary care was not available in our study, and there was possible misclassification in the diagnosis of CVD. However, the validity of CVD diagnoses in the DNPR was high, with the positive predictive values exceeding 90% for most common CVDs.^48^ The death of a sibling was unlikely to impact the recording of CVD in the registers, suggesting that the estimated effect might be biased toward the null due to potential misclassification. Third, Denmark’s well-developed universal welfare system may limit the generalizability of our findings to countries with different health care systems and social contexts.^34^ Fourth, we lacked data on psychosocial resources, including the quality of the relationship with deceased siblings, perceived social support, and participation in psychotherapy or bereavement support groups for the bereaved siblings,^49^ which may affect an individuals’ ability to cope with the loss of a sibling.

## Conclusions

In this cohort study, sibling death in childhood and early adulthood was associated with increased risks of overall and most type-specific early-onset CVDs, and the strengths of these associations varied by cause of death and age difference between sibling pairs. The risk was the highest shortly after the bereavement, especially for adolescents, but persisted in the long run. The findings highlight the need for extra attention and both social and mental support to bereaved siblings to reduce CVD risk later in life.

## Notes

Supplement 1. **eAppendix 1.** Detailed Description of Registers Used in This Study **eAppendix 2.** Detailed Description of Covariates and Multiple Imputation **eTable 1.** Definition of Cardiovascular Disease **eTable 2.** Proportion of Missing Data in Each Baseline Variable **eTable 3.** Baseline Characteristics of the Study Population After Imputation **eTable 4.** Associations Between Sibling Death and CVD by Different Exposure Age and Different Time Since Sibling’s Death **eTable 5.** Associations Between Sibling Death and Type-Specific CVD Stratified on the Cause of Sibling Death **eTable 6.** Mediation Analysis of Mental Disorders on Sibling Death and CVD **eTable 7.** Associations Between Sibling Death and CVD by Sex **eTable 8.** Full Sibling Analyses for the Associations Between Sibling Death and CVD **eTable 9.** Subanalyses for the Associations Between Sibling Death and Overall CVD **eFigure 1.** Flowchart of the Study Participants **eFigure 2.** Directed Acyclic Graph (DAG) Used to Select Covariates for Confounding Control **eFigure 3. **The Log-Minus-Log Survival Curve for Sibling Death and Overall CVD

Supplement 2.
Data Sharing Statement

## References

[1] Timmis A, Vardas P, Townsend N, ; Atlas Writing Group, European Society of Cardiology. European Society of Cardiology: cardiovascular disease statistics 2021. Eur Heart J. 2022;43(8):716-799. doi:10.1093/eurheartj/ehab892 35016208

[2] Andersson C, Vasan RS. Epidemiology of cardiovascular disease in young individuals. Nat Rev Cardiol. 2018;15(4):230-240. doi:10.1038/nrcardio.2017.154 29022571

[3] Roth GA, Mensah GA, Johnson CO, ; GBD-NHLBI-JACC Global Burden of Cardiovascular Diseases Writing Group. Global burden of cardiovascular diseases and risk factors, 1990-2019: update from the GBD 2019 Study. J Am Coll Cardiol. 2020;76(25):2982-3021. doi:10.1016/j.jacc.2020.11.010 33309175 PMC7755038

[4] Yu Y, Arah OA, Liew Z, . Maternal diabetes during pregnancy and early onset of cardiovascular disease in offspring: population based cohort study with 40 years of follow-up. BMJ. 2019;367:l6398. doi:10.1136/bmj.l6398 31801789 PMC6891797

[5] Liu J, Bu X, Wei L, . Global burden of cardiovascular diseases attributable to hypertension in young adults from 1990 to 2019. J Hypertens. 2021;39(12):2488-2496. doi:10.1097/HJH.0000000000002958 34269332

[6] Stroebe M, Schut H, Stroebe W. Health outcomes of bereavement. Lancet. 2007;370(9603):1960-1973. doi:10.1016/S0140-6736(07)61816-9 18068517

[7] Buckley T, McKinley S, Tofler G, Bartrop R. Cardiovascular risk in early bereavement: a literature review and proposed mechanisms. Int J Nurs Stud. 2010;47(2):229-238. doi:10.1016/j.ijnurstu.2009.06.010 19665709

[8] Carey IM, Shah SM, DeWilde S, Harris T, Victor CR, Cook DG. Increased risk of acute cardiovascular events after partner bereavement: a matched cohort study. JAMA Intern Med. 2014;174(4):598-605. doi:10.1001/jamainternmed.2013.14558 24566983

[9] Wei D, Olofsson T, Chen H, . Death of a child and the risk of atrial fibrillation: a nationwide cohort study in Sweden. Eur Heart J. 2021;42(15):1489-1495. doi:10.1093/eurheartj/ehaa1084 33515041 PMC8046501

[10] Wei D, Janszky I, Fang F, . Death of an offspring and parental risk of ischemic heart diseases: a population-based cohort study. PLoS Med. 2021;18(9):e1003790. doi:10.1371/journal.pmed.1003790 34587153 PMC8480908

[11] Wei D, Li J, Chen H, . Death of a child and the risk of stroke: a binational cohort study from Denmark and Sweden. Neurology. 2022;98(11):e1104-e1113. doi:10.1212/WNL.0000000000013263 34996877

[12] Chen H, Li J, Wei D, . Death of a parent and the risk of ischemic heart disease and stroke in Denmark and Sweden. JAMA Netw Open. 2022;5(6):e2218178. doi:10.1001/jamanetworkopen.2022.18178 35731515 PMC9218848

[13] Li J, Hansen D, Mortensen PB, Olsen J. Myocardial infarction in parents who lost a child: a nationwide prospective cohort study in Denmark. Circulation. 2002;106(13):1634-1639. doi:10.1161/01.CIR.0000031569.45667.58 12270855

[14] Mostofsky E, Maclure M, Sherwood JB, Tofler GH, Muller JE, Mittleman MA. Risk of acute myocardial infarction after the death of a significant person in one’s life: the Determinants of Myocardial Infarction Onset Study. Circulation. 2012;125(3):491-496. doi:10.1161/CIRCULATIONAHA.111.061770 22230481 PMC3397171

[15] Wei D, Li J, Janszky I, . Death of a child and the risk of heart failure: a population-based cohort study from Denmark and Sweden. Eur J Heart Fail. 2022;24(1):181-189. doi:10.1002/ejhf.2372 34693593

[16] Bidulka P, Vestergaard SV, Hlupeni A, . Adverse outcomes after partner bereavement in people with reduced kidney function: parallel cohort studies in England and Denmark. PLoS One. 2021;16(9):e0257255. doi:10.1371/journal.pone.0257255 34555018 PMC8460004

[17] Chen H, Wei D, Janszky I, Dahlström U, Rostila M, László KD. Bereavement and prognosis in heart failure: a Swedish cohort study. JACC Heart Fail. 2022;10(10):753-764. doi:10.1016/j.jchf.2022.05.005 36175061

[18] Bengtsson J, Elsenburg LK, Andersen GS, Larsen ML, Rieckmann A, Rod NH. Childhood adversity and cardiovascular disease in early adulthood: a Danish cohort study. Eur Heart J. 2023;44(7):586-593. doi:10.1093/eurheartj/ehac607 36375818

[19] Aalbaek FS, Graff S, Vestergaard M. Risk of stroke after bereavement-a systematic literature review. Acta Neurol Scand. 2017;136(4):293-297. doi:10.1111/ane.12736 28220473

[20] McHale SM, Updegraff KA, Whiteman SD. Sibling relationships and influences in childhood and adolescence. J Marriage Fam. 2012;74(5):913-930. doi:10.1111/j.1741-3737.2012.01011.x 24653527 PMC3956653

[21] Robinson L, Mahon MM. Sibling bereavement: a concept analysis. Death Stud. 1997;21(5):477-499. doi:10.1080/074811897201831 10175164

[22] Segal NL, Wilson SM, Bouchard TJ, Gitlin DG. Comparative Grief experiences of bereaved twins and other bereaved relatives. Pers Individ Dif. 1995;18(4):511-524. doi:10.1016/0191-8869(94)00174-Q

[23] Rostila M, Saarela J, Kawachi I. Mortality from myocardial infarction after the death of a sibling: a nationwide follow-up study from Sweden. J Am Heart Assoc. 2013;2(2):e000046. doi:10.1161/JAHA.112.000046 23537803 PMC3647267

[24] Rostila M, Saarela J, Kawachi I. Fatal stroke after the death of a sibling: a nationwide follow-up study from Sweden. PLoS One. 2013;8(2):e56994. doi:10.1371/journal.pone.0056994 23451131 PMC3579925

[25] Schorr L, Burger A, Hochner H, . Mortality, cancer incidence, and survival in parents after bereavement. Ann Epidemiol. 2016;26(2):115-121. doi:10.1016/j.annepidem.2015.12.008 26809234

[26] Wang MT, Degol JL, Amemiya JL. Older siblings as academic socialization agents for younger siblings: developmental pathways across adolescence. J Youth Adolesc. 2019;48(6):1218-1233. doi:10.1007/s10964-019-01005-2 30903366

[27] Melhem NM, Porta G, Shamseddeen W, Walker Payne M, Brent DA. Grief in children and adolescents bereaved by sudden parental death. Arch Gen Psychiatry. 2011;68(9):911-919. doi:10.1001/archgenpsychiatry.2011.101 21893658 PMC4017669

[28] Rostila M, Berg L, Saarela J, Kawachi I, Hjern A. Experience of sibling death in childhood and risk of death in adulthood: a national cohort study from Sweden. Am J Epidemiol. 2017;185(12):1247-1254. doi:10.1093/aje/kww126 28472250

[29] Yu Y, Liew Z, Cnattingius S, . Association of mortality with the death of a sibling in childhood. JAMA Pediatr. 2017;171(6):538-545. doi:10.1001/jamapediatrics.2017.0197 28437534 PMC5540009

[30] Rostila M, Saarela J, Kawachi I. The forgotten griever: a nationwide follow-up study of mortality subsequent to the death of a sibling. Am J Epidemiol. 2012;176(4):338-346. doi:10.1093/aje/kws163 22814369

[31] Song H, Shang Y, Fang F, . Mortality among twin individuals exposed to loss of a co-twin. Int J Epidemiol. 2023;52(2):600-610. doi:10.1093/ije/dyac145 35849345 PMC10114119

[32] Rostila M, Saarela J, Kawachi I. Suicide following the death of a sibling: a nationwide follow-up study from Sweden. BMJ Open. 2013;3(4):e002618. doi:10.1136/bmjopen-2013-002618 23624991 PMC3641510

[33] Schmidt M, Pedersen L, Sørensen HT. The Danish Civil Registration System as a tool in epidemiology. Eur J Epidemiol. 2014;29(8):541-549. doi:10.1007/s10654-014-9930-3 24965263

[34] Schmidt M, Schmidt SAJ, Adelborg K, . The Danish health care system and epidemiological research: from health care contacts to database records. Clin Epidemiol. 2019;11:563-591. doi:10.2147/CLEP.S179083 31372058 PMC6634267

[35] Palinski W. Effect of maternal cardiovascular conditions and risk factors on offspring cardiovascular disease. Circulation. 2014;129(20):2066-2077. doi:10.1161/CIRCULATIONAHA.113.001805 24842934 PMC4053195

[36] White IR, Royston P, Wood AM. Multiple imputation using chained equations: Issues and guidance for practice. Stat Med. 2011;30(4):377-399. doi:10.1002/sim.4067 21225900

[37] Lee EW, Wei L-J, Amato DA, Leurgans SE. Cox-type regression analysis for large numbers of small groups of correlated failure time observations. In: Klein JP, Goel PK, eds. Survival Analysis: State of the Art. Springer; 1992:234-247.

[38] Brotman DJ, Golden SH, Wittstein IS. The cardiovascular toll of stress. Lancet. 2007;370(9592):1089-1100. doi:10.1016/S0140-6736(07)61305-1 17822755

[39] Kivimäki M, Steptoe A. Effects of stress on the development and progression of cardiovascular disease. Nat Rev Cardiol. 2018;15(4):215-229. doi:10.1038/nrcardio.2017.189 29213140

[40] McEwen BS. Protective and damaging effects of stress mediators. N Engl J Med. 1998;338(3):171-179. doi:10.1056/NEJM199801153380307 9428819

[41] Neugarten BL. Time, age, and the life cycle. Am J Psychiatry. 1979;136(7):887-894. doi:10.1176/ajp.136.7.887 453348

[42] Reimelt C, Wolff N, Hölling H, . Siblings and birth order—are they important for the occurrence of ADHD? J Atten Disord. 2021;25(1):81-90. doi:10.1177/1087054718770020 29720025

[43] Pitman RK, Orr SP, Shalev AY, Metzger LJ, Mellman TA. Psychophysiological alterations in post-traumatic stress disorder. Semin Clin Neuropsychiatry. 1999;4(4):234-241. doi:10.153/SCNP00400234 10553028

[44] Haj-Mirzaian A, Ramezanzadeh K, Shariatzadeh S, . Role of hypothalamic-pituitary adrenal-axis, toll-like receptors, and macrophage polarization in pre-atherosclerotic changes induced by social isolation stress in mice. Sci Rep. 2021;11(1):19091. doi:10.1038/s41598-021-98276-2 34580342 PMC8476494

[45] Shah SM, Carey IM, Harris T, Dewilde S, Victor CR, Cook DG. Impact of partner bereavement on quality of cardiovascular disease management. Circulation. 2013;128(25):2745-2753. doi:10.1161/CIRCULATIONAHA.113.004122 24255060

[46] Merlevede E, Spooren D, Henderick H, . Perceptions, needs and mourning reactions of bereaved relatives confronted with a sudden unexpected death. Resuscitation. 2004;61(3):341-348. doi:10.1016/j.resuscitation.2004.01.024 15172714

[47] Buckley T, Bartrop R, McKinley S, . Prospective study of early bereavement on psychological and behavioural cardiac risk factors. Intern Med J. 2009;39(6):370-378. doi:10.1111/j.1445-5994.2008.01879.x 19460057

[48] Sundbøll J, Adelborg K, Munch T, . Positive predictive value of cardiovascular diagnoses in the Danish National Patient Registry: a validation study. BMJ Open. 2016;6(11):e012832. doi:10.1136/bmjopen-2016-012832 27864249 PMC5129042

[49] Warnick AL. Supporting youth grieving the dying or death of a sibling or parent: considerations for parents, professionals, and communities. Curr Opin Support Palliat Care. 2015;9(1):58-63. doi:10.1097/SPC.0000000000000115 25581448

