# Supplementary material for: Sibling Death in Childhood and Early Adulthood and Risk of Early-Onset Cardiovascular Disease
Source: JAMA Netw Open. 2024 Jan 8;7(1):e2350814. doi: 10.1001/jamanetworkopen.2023.50814 (PMC10774991; doi:10.1001/jamanetworkopen.2023.50814)
Supplement: Supplement 1. — eAppendix 1. Detailed Description of Registers Used in This Study eAppendix 2. Detailed Description of Covariates and Multiple Imputation eTable 1. Definition of Cardiovascular Disease eTable 2. Proportion of Missing Data in Each Baseline Variable eTable 3. Baseline Characteristics of the Study Population After Imputation eTable 4. Associations Between Sibling Death and CVD by Different Exposure Age and Different Time Since Sibling’s Death eTable 5. Associations Between Sibling Death and Type-Specific CVD Stratified on the Cause of Sibling Death eTable 6. Mediation Analysis of Mental Disorders on Sibling Death and CVD eTable 7. Associations Between Sibling Death and CVD by Sex eTable 8. Full Sibling Analyses for the Associations Between Sibling Death and CVD eTable 9. Subanalyses for the Associations Between Sibling Death and Overall CVD eFigure 1. Flowchart of the Study Participants eFigure 2. Directed Acyclic Graph (DAG) Used to Select Covariates for Confounding Control eFigure 3. The Log-Minus-Log Survival Curve for Sibling Death and Overall CVD [file jamanetwopen-e2350814-s001.pdf]

# Supplemental Online Content

Huang C, Peng J, Lee PY, et al. Sibling death in childhood and early adulthood and risk of early-onset cardiovascular disease. *JAMA Netw Open.* 2024;7(1):e2350814. doi:10.1001/jamanetworkopen.2023.50814

**eAppendix 1.** Detailed Description of Registers Used in This Study

**eAppendix 2.** Detailed Description of Covariates and Multiple Imputation

**eTable 1.** Definition of Cardiovascular Disease

**eTable 2.** Proportion of Missing Data in Each Baseline Variable

**eTable 3.** Baseline Characteristics of the Study Population After Imputation

**eTable 4.** Associations Between Sibling Death and CVD by Different Exposure Age and Different Time Since Sibling's Death

**eTable 5.** Associations Between Sibling Death and Type-Specific CVD Stratified on the Cause of Sibling Death

**eTable 6.** Mediation Analysis of Mental Disorders on Sibling Death and CVD

**eTable 7.** Associations Between Sibling Death and CVD by Sex

**eTable 8.** Full Sibling Analyses for the Associations Between Sibling Death and CVD

**eTable 9.** Subanalyses for the Associations Between Sibling Death and Overall CVD

**eFigure 1.** Flowchart of the Study Participants

**eFigure 2.** Directed Acyclic Graph (DAG) Used to Select Covariates for Confounding Control

**eFigure 3.** The Log-Minus-Log Survival Curve for Sibling Death and Overall CVD

This supplemental material has been provided by the authors to give readers additional information about their work.

© 2024 Huang C et al. *JAMA Network Open.*

**eAppendix 1. Detailed Description of Registers Used in This Study**

Denmark has been providing public welfare including universal health care, education, student assistance, disability pensions and unemployment insurance through tax-funded services. There are population-based health databases in Denmark that regularly collect high-quality data including individual medical data and all live births and new residents in Denmark are assigned a unique 10-digit individual personal identification number (Central Personal Register number, CPR) since the 1960s. Information across different national registers are linked by the CPR number.[1, 2]

**The Danish Registry of Causes of Death**

The Danish Registry of Causes of Death has been computerized since 1970 and the non-electronic data on deaths has been collected since 1924.[3] This system has been collecting date of death, and immediate cause, underlying cause and contributory causes of death by law in Denmark. The ICD-8 was used to classify the causes of death during 1970-1993 and ICD-10 from 1994.

**The Danish National Patient Register (DNPR)**

The Danish National Patient Register (DNPR) was established in 1977 and collects data from inpatient, outpatient, and emergency hospital record (each visit for one record in register).[4] Information includes date of contact, primary and secondary discharge diagnoses, date and procedures of surgery, certain treatment in hospitals, and other hospital related information. Diagnoses are classified according to the ICD-8 codes (International Classification of Disease codes, 8th revision) and ICD-10 codes thereafter.

**The Danish Medical Birth Registry (MBR)**

© 2024 Huang C et al. *JAMA Network Open.*

The Danish Medical Birth Registry (MBR) includes CPR number of new births, mothers, and fathers, as well as information on date of birth, birth characteristics such as sex, birth weight, gestational age, maternal characteristics such as maternal pre-pregnancy body mass index, maternal smoking during pregnancy. MBR was established in 1968 and has been computerized since 1973.[5]

**The Danish Integrated Database for Longitudinal Labour Market Research**

The Danish Integrated Database for Longitudinal Labour Market Research was established in 1981 and contains labour market and socioeconomic data.

**The Danish Civil Registration System (CRS)**

The Danish Civil Registration System (CRS) has been established since 1968, and collects information on date of birth, emigration, and immigration, sex, and other vital status in a daily basis.[2]

© 2024 Huang C et al. *JAMA Network Open.*

**eAppendix 2. Detailed Description of Covariates and Multiple Imputation**

Information on maternal and birth characteristics were retrieved from the Danish Medical Birth Registry (MBR),[5] whereas socioeconomic factors were retrieved from the Danish Integrated Database for Longitudinal Labor Market Research, the Danish Civil Registry System (CRS).[2] This included: preterm birth (<37, ⩾37weeks, or unknown), birth weight (<2500, 2500-3249, 3250-3999, ⩾4000 g, or unknown), singleton birth (yes or no), parity (1, 2, or ⩾3), Apgar score at 5 minutes (0-6,7-9, 10, or unknown), maternal age (⩽20, 20-25, 25-30, 30-35 or ⩾35 years), maternal education (0-9, 10-14, ⩾15 years, or unknown), maternal smoking during pregnancy (yes or no), sex of child (male or female), and calendar period (1980 and before, a 10-year interval during 1980-2009, 2010 and after), parental history of CVD (yes or no) before child birth, maternal hypertensive disorder of pregnancy (yes or no), and parental history of diabetes (yes or no) before child birth.

We imputed missing variables using the fully conditional specification (FCS) method in which logistic regression was used for categorical variables and predictive mean matching for continuous variables.[6] The imputation model included the following variables: preterm birth (<37, ≥37weeks, or unknown), birth weight (<2500, 2500-3249, 3250-3999, ≥4000 g, or unknown), singleton birth (yes or no), parity (1, 2, or ≥3), Apgar score at 5 minutes (0-6,7-9, 10, or unknown), maternal age (≤20, 20-25, 25-30, 30-35, or ≥35 years), maternal education (0-9, 10-14, ≥15 years, or unknown), maternal smoking during pregnancy (yes or no), sex of child (male or female), and calendar period (1980 and before, a 10-year interval during 1980-2009, 2010 and after), parental history of CVD (yes or no) before child birth, maternal hypertensive disorder of pregnancy (yes or no), and parental history of diabetes (yes or no)

© 2024 Huang C et al. *JAMA Network Open.*

before child birth. As the data on birth weight, maternal educational level was available since 1979, and maternal smoking data during pregnancy was available from 1991, the imputation model for these variables was only restricted to individuals born since the year they became available.

**References**


1. Schmidt M, Schmidt SAJ, Adelborg K, et al. The Danish health care system and epidemiological research: from health care contacts to database records. Clin Epidemiol. 2019;11:563-591. doi:10.2147/CLEP.S179083
2. Schmidt M, Pedersen L, Sørensen HT. The Danish Civil Registration System as a tool in epidemiology. Eur J Epidemiol. Aug 2014;29(8):541-9. doi:10.1007/s10654-014-9930-3
3. Helweg-Larsen K. The Danish Register of Causes of Death. Scand J Public Health. Jul 2011;39(7 Suppl):26-9. doi:10.1177/1403494811399958
4. Hess DT, Jr. The Danish National Patient Register. Surg Obes Relat Dis. Feb 2016;12(2):304. doi:10.1016/j.soard.2015.11.001
5. Bliddal M, Broe A, Pottegård A, Olsen J, Langhoff-Roos J. The Danish Medical Birth Register. Eur J Epidemiol. Jan 2018;33(1):27-36. doi:10.1007/s10654-018-0356-1
6. White IR, Royston P, Wood AM. Multiple imputation using chained equations: Issues and guidance for practice. Stat Med, 2011;30:377-399. doi: 10.1002/sim.4067


© 2024 Huang C et al. *JAMA Network Open.*

**eTable 1. Definition of Cardiovascular Disease[a]**

| | ICD-8 | ICD-10 | Procedure/Surgery Codes |
|---|---|---|---|
| Overall cardiovascular disease (CVD) | 390-444.1, 444.3-458, 782.4 | I00-I99 | 30350, 30354, 30240, KFNG, KFNF 30009, 30019, 30029, 30039, 30049, 30059, 30069, 30079, 30089, 30099, 30109, 30119, 30120, 30129, 30139, 30149, 30159, 30169, 30179, 30189, 30199, 30200, KFNA-KFNE, KFNH20 |
| Ischemic heart disease | 410-414 | I20-I25 | |
| Myocardial infarction | 410 | I21 | |
| Cerebrovascular disease | 430-438 | I60-I69 | |
| Stroke | 430-436 | I61-I64 | |
| Ischemic stroke | 433-434 | I63-I64 | |
| Heart failure | 427.0, 427.1, 782.4 | I110, I130, I132, I50 | |
| Atrial fibrillation | 427.93, 427.94 | I48 | |
| Hypertensive disease | 400-404 | I10-I15 | |
| Deep vein thrombosis | 451.00 | I80.1-I80.3 | |
| Pulmonary embolism | 450.99 | I26 | |
| Rheumatic heart disease | 391, 393-398 | I01, I05-09 | |
| Peripheral artery disease | 443.89-443.99 | I73.9 | |

[a] The information on cardiovascular disease is from the Danish National Patient Registry or the Danish Register of Cause of Death.

© 2024 Huang C et al. *JAMA Network Open.*

**eTable 2. Proportion of Missing Data in Each Baseline Variable[a]**

| Variables | Number of missing data | Proportion of missing data |
|---|---|---|
| Sex | / | / |
| Preterm Birth | 68949 | 3.3% |
| Birth Weight, g [b] | 24189 | 1.2% |
| Singleton | / | / |
| Parity | / | / |
| Apgar score at 5 minutes | 40968 | 2.0% |
| Maternal age at birth, y | / | / |
| Maternal education at birth, y [b] | 22964 | 1.1% |
| Maternal smoke during pregnancy [c] | 61493 | 4.2% |
| Parents with CVD history | / | / |
| Maternal gestational hypertension | / | / |
| Parents diabetes history | / | / |

[a] Expressed as frequency (percentage).

[b] Birth weight, maternal educational level data were available in Denmark from 1979 to 2018.

[c] Maternal smoking data during pregnancy was available in Denmark from 1991 to 2018.

© 2024 Huang C et al. *JAMA Network Open.*

**eTable 3. Baseline Characteristics of the Study Population After Imputation**

| Variables | Study cohort, No. (%) [a] | | |
|---|---|---|---|
| | Exposed (N=22,968) | Unexposed (N=2,075,691) | Total (N=2,098,659) |
| Sex | | | |
| Male | 11751(51.2) | 1064918(51.3) | 1076669(51.3) |
| Female | 11217(48.8) | 1010773(48.7) | 1021990(48.7) |
| Preterm Birth | | | |
| No | 21337(92.9) | 1960376(94.4) | 1981713(94.4) |
| Yes | 1631(7.1) | 115315(5.6) | 116946(5.6) |
| Birth Weight, g | | | |
| <2500 | 1513(7.0) | 97819(4.8) | 99332(4.8) |
| 2500-3249 | 6739(31.0) | 528193(26.0) | 534932(26.1) |
| 3250-3999 | 10273(47.3) | 1045928(51.5) | 1056201(51.5) |
| ≥4000g | 3214(14.8) | 357149(17.6) | 360363(17.6) |
| Singleton | | | |
| No | 679(3.0) | 76128(3.7) | 76807(3.7) |
| Yes | 22289(97.0) | 1999563(96.3) | 2021852(96.3) |
| Parity | | | |
| 1 | 8985(39.1) | 821536(39.6) | 830521(39.6) |
| 2 | 7266(31.6) | 855718(41.2) | 862984(41.1) |
| ≥3 | 6717(29.2) | 398437(19.2) | 405154(19.3) |
| Apgar score at 5 minutes | | | |
| 0-6 | 172(0.7) | 14389(0.7) | 14561(0.7) |
| 7-9 | 1380(6.0) | 125070(6.0) | 126450(6.0) |
| 10 | 21416(93.2) | 1936232(93.3) | 1957648(93.3) |
| Maternal age at birth, y | | | |
| 15-19 | 1304(5.7) | 46359(2.2) | 47663(2.3) |
| 20-24 | 6280(27.3) | 362676(17.5) | 368956(17.6) |
| 25-29 | 8065(35.1) | 766762(36.9) | 774827(36.9) |
| 30-34 | 5066(22.1) | 629473(30.3) | 634539(30.2) |
| ≥35 | 2253(9.8) | 270421(13.0) | 272674(13.0) |
| Maternal education at birth, y | | | |
| 0-9 | 10238(47.1) | 525305(25.9) | 535543(26.1) |
| 45213 | 7831(36.0) | 892550(44.0) | 900381(43.9) |
| ≥15 | 3670(16.9) | 611234(30.1) | 614904(30.0) |
| Maternal smoke during pregnancy | | | |
| No | 6209(67) | 1184848(80.9) | 1191057(80.9) |
| Yes | 3059(33) | 279022(19.1) | 282081(19.1) |
| Parents with CVD history | | | |
| No | 22251(96.9) | 1969194(94.9) | 1991445(94.9) |

© 2024 Huang C et al. *JAMA Network Open.*

| | | | |
|---|---|---|---|
| Yes | 717(3.1) | 106497(5.1) | 107214(5.1) |
| Maternal gestational hypertension | | | |
| No | 21808(94.9) | 1962207(94.5) | 1984015(94.5) |
| Yes | 1160(5.1) | 113484(5.5) | 114644(5.5) |
| Parents diabetes history | | | |
| No | 22694(98.8) | 2040158(98.3) | 2062852(98.3) |
| Yes | 274(1.2) | 35533(1.7) | 35807(1.7) |

Abbreviations: CVD, cardiovascular disease.

[a] Expressed as frequency (percentage).

© 2024 Huang C et al. *JAMA Network Open.*

**eTable 4. Associations Between Sibling Death and CVD by Different Exposure Age and Different Time Since Sibling's Death**

| Exposure age (yrs) | Time since sibling's death, yrs | Exposure | No. of observations | No. of CVD cases | Rate per 1,000 person-years | Crude HR (95%CI) | Adjusted HR (95%CI) [a] |
|---|---|---|---|---|---|---|---|
| 0-5 | < 1 | unexposed | 9329180 | 5700 | 0.61 | 1.0(ref) | 1.0(ref) |
| | | exposed | 7250 | 9 | 1.24 | 1.86(0.97-3.59) | 2.23(1.16-4.29) |
| | 1-4 | unexposed | 15891416 | 9731 | 0.61 | 1.0(ref) | 1.0(ref) |
| | | exposed | 27716 | 13 | 0.47 | 0.78(0.45-1.35) | 0.89(0.52-1.54) |
| | 5-9 | unexposed | 17149203 | 14716 | 0.86 | 1.0(ref) | 1.0(ref) |
| | | exposed | 31837 | 25 | 0.79 | 1.11(0.75-1.64) | 1.13(0.76-1.67) |
| | 10-14 | unexposed | 14606190 | 24825 | 1.7 | 1.0(ref) | 1.0(ref) |
| | | exposed | 28294 | 45 | 1.59 | 1.08(0.81-1.45) | 1.01(0.76-1.36) |
| | ≥15 | unexposed | 16299269 | 60792 | 3.73 | 1.0(ref) | 1.0(ref) |
| | | exposed | 60439 | 302 | 5 | 1.22(1.09-1.36) | 1.13(1.01-1.26) |
| 6-11 | < 1 | unexposed | 11294522 | 7645 | 0.68 | 1.0(ref) | 1.0(ref) |
| | | exposed [b] | 4583 | - | 0.87 | 1.48(0.55-3.93) | 1.59(0.59-4.24) |
| | 1-4 | unexposed | 15048374 | 15318 | 1.02 | 1.0(ref) | 1.0(ref) |
| | | exposed | 17422 | 19 | 1.09 | 1.30(0.83-2.04) | 1.34(0.86-2.11) |
| | 5-9 | unexposed | 13999215 | 26980 | 1.93 | 1.0(ref) | 1.0(ref) |
| | | exposed | 19530 | 42 | 2.15 | 1.24(0.91-1.67) | 1.19(0.88-1.61) |
| | 10-14 | unexposed | 10769248 | 34579 | 3.21 | 1.0(ref) | 1.0(ref) |
| | | exposed | 16272 | 47 | 2.89 | 0.94(0.71-1.25) | 0.87(0.66-1.16) |
| | ≥15 | unexposed | 9154791 | 43456 | 4.75 | 1.0(ref) | 1.0(ref) |
| | | exposed | 24848 | 155 | 6.24 | 1.24(1.06-1.45) | 1.15(0.98-1.34) |
| 12-17 | < 1 | unexposed | 9327333 | 15957 | 1.71 | 1.0(ref) | 1.0(ref) |
| | | exposed | 3604 | 17 | 4.72 | 3.48(2.16-5.60) | 3.60(2.23-5.80) |
| | 1-4 | unexposed | 11880093 | 27661 | 2.33 | 1.0(ref) | 1.0(ref) |
| | | exposed | 13247 | 46 | 3.47 | 1.62(1.21-2.16) | 1.57(1.17-2.10) |
| | 5-9 | unexposed | 10102416 | 34904 | 3.46 | 1.0(ref) | 1.0(ref) |
| | | exposed | 13859 | 59 | 4.26 | 1.29(1.00-1.67) | 1.21(0.94-1.56) |
| | 10-14 | unexposed | 6843111 | 32113 | 4.69 | 1.0(ref) | 1.0(ref) |
| | | exposed | 9873 | 51 | 5.17 | 1.14(0.87-1.50) | 1.06(0.80-1.39) |
| | ≥15 | unexposed | 4195295 | 23850 | 5.68 | 1.0(ref) | 1.0(ref) |
| | | exposed | 8810 | 54 | 6.13 | 1.06(0.81-1.38) | 1.01(0.77-1.31) |
| ≥18 | < 1 | unexposed | 12463929 | 53011 | 4.25 | 1.0(ref) | 1.0(ref) |
| | | exposed | 7531 | 67 | 8.9 | 2.86(2.24-3.64) | 2.52(1.98-3.21) |
| | 1-4 | unexposed | 11300764 | 49922 | 4.42 | 1.0(ref) | 1.0(ref) |
| | | exposed | 23048 | 143 | 6.2 | 1.65(1.40-1.95) | 1.45(1.23-1.71) |
| | 5-9 | unexposed | 7252818 | 36842 | 5.08 | 1.0(ref) | 1.0(ref) |
| | | exposed | 17036 | 116 | 6.81 | 1.42(1.18-1.70) | 1.26(1.05-1.51) |
| | 10-14 | unexposed | 3583436 | 20940 | 5.84 | 1.0(ref) | 1.0(ref) |
| | | exposed | 7566 | 61 | 8.06 | 1.37(1.07-1.77) | 1.24(0.97-1.60) |

© 2024 Huang C et al. *JAMA Network Open.*

| | | | | | | |
|---|---|---|---|---|---|---|
| ≥15 | unexposed | 1329664 | 8038 | 6.05 | 1.0(ref) | 1.0(ref) |
| | exposed | 2230 | 11 | 4.93 | 0.83(0.46-1.50) | 0.77(0.42-1.39) |

[a] Adjusted for preterm birth, birth weight, singleton birth, parity, Apgar score at 5 minutes, maternal age, maternal education, maternal smoking during pregnancy, sex of child, and calendar period, maternal hypertensive disorder of pregnancy, parental history of CVD before child birth, parental history of diabetes before child birth.

[b] <6 cases are not allowed to report due to data protection in Denmark.

© 2024 Huang C et al. *JAMA Network Open.*

**eTable 5. Associations Between Sibling Death and Type-Specific CVD Stratified on the Cause of Sibling Death**

| Outcome and exposure | No. of observations | No. of CVD cases | Rate per 1,000 person-years | cHR (95%CI) | aHR (95%CI) [a] |
|---|---|---|---|---|---|
| **Ischemic heart disease** | | | | | |
| Unexposed | 2080860 | 2681 | 0.07 | 1.0(ref) | 1.0(ref) |
| Exposed to sibling death due to CVD | 926 | 8 | 0.96 | 4.96(2.48-9.93) | 4.62(2.31-9.26) |
| Exposed to sibling death due to non-CVD cause | 22519 | 72 | 0.21 | 1.59(1.26-2.01) | 1.42(1.12-1.79) |
| **Myocardial infarction** | | | | | |
| Unexposed | 2080887 | 698 | 0.02 | 1.0(ref) | 1.0(ref) |
| Exposed to sibling death due to CVD [b] | 926 | - | 0.36 | 5.72(1.84-17.78) | 5.35(1.72-16.64) |
| Exposed to sibling death due to non-CVD cause | 22529 | 23 | 0.07 | 1.73(1.14-2.62) | 1.53(1.01-2.31) |
| **Cerebrovascular disease** | | | | | |
| Unexposed | 2080699 | 2817 | 0.07 | 1.0(ref) | 1.0(ref) |
| Exposed to sibling death due to CVD | 925 | 6 | 0.72 | 4.48(2.01-9.99) | 4.25(1.91-9.46) |
| Exposed to sibling death due to non-CVD cause | 22514 | 55 | 0.16 | 1.33(1.02-1.73) | 1.25(0.95-1.63) |
| **Stroke** | | | | | |
| Unexposed | 2080655 | 1060 | 0.03 | 1.0(ref) | 1.0(ref) |
| Exposed to sibling death due to CVD [b] | 927 | - | 0.00 | - | - |
| Exposed to sibling death due to non-CVD cause | 22524 | 21 | 0.06 | 1.65(1.07-2.54) | 1.52(0.98-2.34) |
| **Ischemic stroke** | | | | | |
| Unexposed | 2080640 | 1861 | 0.05 | 1.0(ref) | 1.0(ref) |
| Exposed to sibling death due to CVD [b] | 925 | - | 0.60 | 4.87(2.02-11.72) | 4.61(1.92-11.10) |
| Exposed to sibling death due to non-CVD cause | 22518 | 35 | 0.10 | 1.19(0.85-1.67) | 1.12(0.80-1.56) |
| **Heart failure** | | | | | |
| Unexposed | 2080806 | 890 | 0.02 | 1.0(ref) | 1.0(ref) |
| Exposed to sibling death due to CVD | 923 | 6 | 0.72 | 11.86(5.31-26.49) | 11.53(5.15-25.78) |
| Exposed to sibling death due to non-CVD cause | 22519 | 18 | 0.05 | 1.26(0.79-2.01) | 1.17(0.73-1.86) |
| **Atrial fibrillation** | | | | | |
| Unexposed | 2080862 | 2066 | 0.05 | 1.0(ref) | 1.0(ref) |

© 2024 Huang C et al. *JAMA Network Open.*

| | | | | | |
|---|---|---|---|---|---|
| Exposed to sibling death due to CVD [b] | 925 | - | 0.12 | 0.87(0.12-6.08) | 0.85(0.12-6.07) |
| Exposed to sibling death due to non-CVD cause | 22523 | 32 | 0.09 | 0.94(0.66-1.34) | 0.92(0.65-1.30) |
| **Hypertensive disease** | | | | | |
| Unexposed | 2080698 | 8623 | 0.23 | 1.0(ref) | 1.0(ref) |
| Exposed to sibling death due to CVD | 917 | 12 | 1.44 | 2.48(1.41-4.36) | 2.32(1.32-4.08) |
| Exposed to sibling death due to non-CVD cause | 22491 | 168 | 0.50 | 1.19(1.02-1.39) | 1.11(0.95-1.30) |
| **Deep vein thrombosis** | | | | | |
| Unexposed | 2080882 | 4383 | 0.12 | 1.0(ref) | 1.0(ref) |
| Exposed to sibling death due to CVD [b] | 920 | - | 0.60 | 2.10(0.88-5.05) | 2.01(0.84-4.83) |
| Exposed to sibling death due to non-CVD cause | 22502 | 102 | 0.30 | 1.44(1.18-1.75) | 1.36(1.12-1.66) |
| **Pulmonary embolism** | | | | | |
| Unexposed | 2080847 | 2169 | 0.06 | 1.0(ref) | 1.0(ref) |
| Exposed to sibling death due to CVD [b] | 923 | - | 0.24 | 1.70(0.43-6.81) | 1.59(0.40-6.36) |
| Exposed to sibling death due to non-CVD cause | 22518 | 54 | 0.16 | 1.54(1.18-2.02) | 1.46(1.12-1.92) |
| **Rheumatic heart disease** | | | | | |
| Unexposed | 2080867 | 209 | 0.01 | 1.0(ref) | 1.0(ref) |
| Exposed to sibling death due to CVD [b] | 927 | - | 0.00 | - | - |
| Exposed to sibling death due to non-CVD cause [b] | 22529 | - | 0.01 | 1.66(0.61-4.46) | 1.56(0.58-4.21) |
| **Peripheral artery disease** | | | | | |
| Unexposed | 2080887 | 437 | 0.01 | 1.0(ref) | 1.0(ref) |
| Exposed to sibling death due to CVD [b] | 927 | - | 0.00 | - | - |
| Exposed to sibling death due to non-CVD cause | 22529 | 6 | 0.02 | 0.92(0.41-2.06) | 0.88(0.39-1.98) |

Abbreviations: CVD, cardiovascular disease; cHR, crude hazard ratio; aHR, adjusted hazard ratio.

[a] Adjusted for preterm birth, birth weight, singleton birth, parity, Apgar score at 5 minutes, maternal age, maternal education, maternal smoking during pregnancy, sex of child, and calendar period, maternal hypertensive disorder of pregnancy, parental history of CVD before child birth, parental history of diabetes before child birth.

[b] <6 cases are not allowed to report due to data protection in Denmark.

© 2024 Huang C et al. *JAMA Network Open.*

**eTable 6. Mediation Analysis of Mental Disorders on Sibling Death and CVD**

| **Outcome** | **$HR_{TE}$** | **$HR_{CDE}$** | **$HR_{PE}$** | **Proportion eliminated (%)** |
|---|---|---|---|---|
| Overall CVD | 1.17(1.10-1.24) | 1.13(1.06-1.20) | 1.03(1.01-1.05) | 21.9(4.8-39.2) |

Abbreviations: CVD, cardiovascular disease; HR, hazard ratio.

[a] Adjusted for preterm birth, birth weight, singleton birth, parity, Apgar score at 5 minutes, maternal age, maternal education, maternal smoking during pregnancy, sex of child, and calendar period, maternal hypertensive disorder of pregnancy, parental history of CVD before child birth, parental history of diabetes before child birth. $HR_{PE}$ = ($HR_{TE}$/$HR_{CDE}$). Proportion eliminated = ($HR_{TE}$ - $HR_{CDE}$)/($HR_{TE}$ - 1), only present if the direction of CDE and PE was the same. The boot-strapped CIs for HRPE and proportion eliminated were obtained using 100 replicates.

© 2024 Huang C et al. *JAMA Network Open.*

eTable 7. Associations Between Sibling Death and CVD by Sex

| Outcome and exposure | Males (N=1,076,669) | | | | | Females (N=1,021,990) | | | | |
|---|---|---|---|---|---|---|---|---|---|---|
| | No. of observations | No. of CVD cases | Rate per 1,000 person-years | cHR (95%CI) | aHR (95%CI) [b] | No. of observations | No. of CVD cases | Rate per 1,000 person-years | cHR (95%CI) | aHR (95%CI) [a] |
| **Overall CVD** | | | | | | | | | | |
| Unexposed | 1064918 | 36207 | 1.91 | 1.0(ref) | 1.0(ref) | 1010773 | 40655 | 2.25 | 1.0(ref) | 1.0(ref) |
| Exposed | 11751 | 561 | 3.29 | 1.17(1.08-1.27) | 1.15(1.06-1.25) | 11217 | 725 | 4.45 | 1.21(1.13-1.31) | 1.18(1.10-1.27) |
| **Ischemic heart disease** | | | | | | | | | | |
| Unexposed | 1067873 | 1660 | 0.09 | 1.0(ref) | 1.0(ref) | 1012987 | 1021 | 0.06 | 1.0(ref) | 1.0(ref) |
| Exposed | 11988 | 45 | 0.26 | 1.54(1.15-2.07) | 1.38(1.03-1.86) | 11457 | 35 | 0.21 | 1.97(1.41-2.77) | 1.75(1.24-2.45) |
| **Myocardial infarction** | | | | | | | | | | |
| Unexposed | 1067890 | 505 | 0.03 | 1.0(ref) | 1.0(ref) | 1012997 | 193 | 0.01 | 1.0(ref) | 1.0(ref) |
| Exposed | 11993 | 16 | 0.09 | 1.60(0.97-2.63) | 1.44(0.87-2.37) | 11462 | 10 | 0.06 | 2.63(1.39-4.97) | 2.19(1.16-4.15) |
| **Cerebrovascular disease** | | | | | | | | | | |
| Unexposed | 1067776 | 1379 | 0.07 | 1.0(ref) | 1.0(ref) | 1012923 | 1438 | 0.08 | 1.0(ref) | 1.0(ref) |
| Exposed | 11984 | 30 | 0.17 | 1.48(1.03-2.12) | 1.39(0.97-2.00) | 11455 | 31 | 0.18 | 1.38(0.97-1.97) | 1.30(0.91-1.85) |
| **Stroke** | | | | | | | | | | |
| Unexposed | 1067749 | 636 | 0.03 | 1.0(ref) | 1.0(ref) | 1012906 | 424 | 0.02 | 1.0(ref) | 1.0(ref) |
| Exposed | 11990 | 9 | 0.05 | 1.13(0.58-2.18) | 1.04(0.54-2.02) | 11461 | 12 | 0.07 | 2.33(1.31-4.15) | 2.13(1.20-3.80) |
| **Ischemic stroke** | | | | | | | | | | |
| Unexposed | 1067764 | 833 | 0.04 | 1.0(ref) | 1.0(ref) | 1012876 | 1028 | 0.06 | 1.0(ref) | 1.0(ref) |
| Exposed | 11987 | 19 | 0.11 | 1.41(0.89-2.22) | 1.33(0.84-2.10) | 11456 | 21 | 0.12 | 1.24(0.81-1.92) | 1.16(0.75-1.78) |
| **Heart failure** | | | | | | | | | | |
| Unexposed | 1067847 | 545 | 0.03 | 1.0(ref) | 1.0(ref) | 1012959 | 345 | 0.02 | 1.0(ref) | 1.0(ref) |
| Exposed | 11984 | 11 | 0.06 | 1.18(0.65-2.15) | 1.11(0.61-2.02) | 11458 | 13 | 0.08 | 2.36(1.35-4.11) | 2.16(1.23-3.76) |
| **Atrial fibrillation** | | | | | | | | | | |

© 2024 Huang C et al. *JAMA Network Open.*

| | | | | | | | | | | |
|---|---|---|---|---|---|---|---|---|---|---|
| Unexposed | 1067872 | 1343 | 0.07 | 1.0(ref) | 1.0(ref) | 1012990 | 723 | 0.04 | 1.0(ref) | 1.0(ref) |
| Exposed | 11987 | 19 | 0.11 | 0.81(0.52-1.28) | 0.80(0.51-1.26) | 11461 | 14 | 0.08 | 1.19(0.70-2.01) | 1.14(0.67-1.93) |
| **Hypertensive disease** | | | | | | | | | | |
| Unexposed | 1067770 | 3681 | 0.19 | 1.0(ref) | 1.0(ref) | 1012928 | 4942 | 0.27 | 1.0(ref) | 1.0(ref) |
| Exposed | 11982 | 74 | 0.42 | 1.23(0.98-1.55) | 1.14(0.90-1.44) | 11426 | 106 | 0.63 | 1.24(1.02-1.50) | 1.16(0.96-1.41) |
| **Deep vein thrombosis** | | | | | | | | | | |
| Unexposed | 1067889 | 1354 | 0.07 | 1.0(ref) | 1.0(ref) | 1012993 | 3029 | 0.17 | 1.0(ref) | 1.0(ref) |
| Exposed | 11983 | 41 | 0.23 | 1.75(1.28-2.39) | 1.67(1.22-2.29) | 11439 | 66 | 0.39 | 1.32(1.04-1.69) | 1.24(0.97-1.59) |
| **Pulmonary embolism** | | | | | | | | | | |
| Unexposed | 1067862 | 567 | 0.03 | 1.0(ref) | 1.0(ref) | 1012985 | 1602 | 0.09 | 1.0(ref) | 1.0(ref) |
| Exposed | 11993 | 19 | 0.11 | 1.94(1.23-3.07) | 1.83(1.16-2.89) | 11448 | 37 | 0.22 | 1.40(1.01-1.94) | 1.34(0.96-1.85) |
| **Rheumatic heart disease** | | | | | | | | | | |
| Unexposed | 1067881 | 100 | 0.01 | 1.0(ref) | 1.0(ref) | 1012986 | 109 | 0.01 | 1.0(ref) | 1.0(ref) |
| Exposed [b] | 11995 | - | 0.01 | 0.97(0.13-6.96) | 0.90(0.13-6.52) | 11461 | - | 0.02 | 2.07(0.65-6.53) | 1.95(0.61-6.17) |
| **Peripheral artery disease** | | | | | | | | | | |
| Unexposed | 1067894 | 201 | 0.01 | 1.0(ref) | 1.0(ref) | 1012993 | 236 | 0.01 | 1.0(ref) | 1.0(ref) |
| Exposed [b] | 11995 | - | 0.01 | 0.63(0.16-2.55) | 0.60(0.15-2.43) | 11461 | - | 0.02 | 1.11(0.41-3.00) | 1.10(0.41-2.96) |

Abbreviations: CVD, cardiovascular disease; cHR, crude hazard ratio; aHR, adjusted hazard ratio.

[a] Adjusted for preterm birth, birth weight, singleton birth, parity, Apgar score at 5 minutes, maternal age, maternal education, maternal smoking during pregnancy, sex of child, and calendar period, maternal hypertensive disorder of pregnancy, parental history of CVD before child birth, parental history of diabetes before child birth.

[b] <6 cases are not allowed to report due to data protection in Denmark.

© 2024 Huang C et al. *JAMA Network Open.*

**eTable 8. Full Sibling Analyses for the Associations Between Sibling Death and CVD[a]**

| Outcome and exposure | No. of observations | No. of CVD cases | Rate per 1,000 person-years | cHR (95%CI) | aHR (95%CI) [b] |
|---|---|---|---|---|---|
| **Overall CVD** | | | | | |
| Unexposed | 1884733 | 68790 | 2.03 | 1.0(ref) | 1.0(ref) |
| Exposed | 17389 | 973 | 3.79 | 1.19(1.12-1.27) | 1.17(1.10-1.25) |
| **Ischemic heart disease** | | | | | |
| Unexposed | 1889042 | 2340 | 0.07 | 1.0(ref) | 1.0(ref) |
| Exposed | 17751 | 63 | 0.24 | 1.80(1.40-2.31) | 1.64(1.27-2.10) |
| **Myocardial infarction** | | | | | |
| Unexposed | 1889062 | 607 | 0.02 | 1.0(ref) | 1.0(ref) |
| Exposed | 17760 | 22 | 0.08 | 2.11(1.38-3.23) | 1.91(1.25-2.93) |
| **Cerebrovascular disease** | | | | | |
| Unexposed | 1888899 | 2486 | 0.07 | 1.0(ref) | 1.0(ref) |
| Exposed | 17747 | 42 | 0.16 | 1.32(0.97-1.79) | 1.26(0.93-1.71) |
| **Stroke** | | | | | |
| Unexposed | 1888854 | 958 | 0.03 | 1.0(ref) | 1.0(ref) |
| Exposed | 17757 | 16 | 0.06 | 1.61(0.98-2.64) | 1.50(0.92-2.47) |
| **Ischemic stroke** | | | | | |
| Unexposed | 1888848 | 1625 | 0.05 | 1.0(ref) | 1.0(ref) |
| Exposed | 17752 | 30 | 0.11 | 1.33(0.93-1.91) | 1.27(0.89-1.83) |
| **Heart failure** | | | | | |
| Unexposed | 1888993 | 786 | 0.02 | 1.0(ref) | 1.0(ref) |
| Exposed | 17750 | 20 | 0.08 | 1.79(1.15-2.79) | 1.69(1.08-2.63) |
| **Atrial fibrillation** | | | | | |
| Unexposed | 1889036 | 1799 | 0.05 | 1.0(ref) | 1.0(ref) |
| Exposed | 17753 | 25 | 0.09 | 0.95(0.64-1.41) | 0.94(0.63-1.39) |
| **Hypertensive disease** | | | | | |
| Unexposed | 1888912 | 7739 | 0.22 | 1.0(ref) | 1.0(ref) |
| Exposed | 17724 | 140 | 0.53 | 1.25(1.06-1.48) | 1.19(1.00-1.40) |
| **Deep vein thrombosis** | | | | | |
| Unexposed | 1889059 | 3815 | 0.11 | 1.0(ref) | 1.0(ref) |
| Exposed | 17737 | 72 | 0.27 | 1.32(1.04-1.66) | 1.27(1.01-1.60) |
| **Pulmonary embolism** | | | | | |
| Unexposed | 1889024 | 1903 | 0.06 | 1.0(ref) | 1.0(ref) |
| Exposed | 17751 | 36 | 0.14 | 1.33(0.96-1.85) | 1.29(0.93-1.79) |
| **Rheumatic heart disease** | | | | | |
| Unexposed | 1889044 | 192 | 0.01 | 1.0(ref) | 1.0(ref) |
| Exposed [c] | 17762 | - | 0.01 | 1.54(0.49-4.84) | 1.44(0.46-4.53) |
| **Peripheral artery disease** | | | | | |
| Unexposed | 1889061 | 394 | 0.01 | 1.0(ref) | 1.0(ref) |
| Exposed [c] | 17761 | - | 0.02 | 0.98(0.41-2.38) | 0.97(0.40-2.34) |

Abbreviations: CVD, cardiovascular disease; cHR, crude hazard ratio; aHR, adjusted hazard ratio.

[a] Full sibling analysis included 1,902,122 participants.
[b] Adjusted for preterm birth, birth weight, singleton birth, parity, Apgar score at 5 minutes, maternal age, maternal education, maternal smoking during pregnancy, sex of child, and calendar period, maternal hypertensive disorder of pregnancy, parental history of CVD before child birth, parental history of diabetes before child birth.
[c] <6 cases are not allowed to report due to data protection in Denmark.

© 2024 Huang C et al. *JAMA Network Open.*

eTable 9. Subanalyses for the Associations Between Sibling Death and Overall CVD

| | Exposure | No. of observations | No. of CVD cases | Rate per 1,000 person-years | Crude HR (95%CI) | Adjusted HR (95%CI) [a] |
|---|---|---|---|---|---|---|
| **Additionally adjusted for parental mental disorders** | unexposed | 2075691 | 76862 | 2.08 | 1.0(ref) | 1.0(ref) |
| **(N=2,098,659)** | exposed | 22968 | 1286 | 3.86 | 1.19(1.13-1.26) | 1.17(1.11-1.24) |
| **Adjusted for minimal adjustment sets** | unexposed | 2075691 | 76862 | 2.08 | 1.0(ref) | 1.0(ref) |
| **(N=2,098,659)** | exposed | 22968 | 1286 | 3.86 | 1.19(1.13-1.26) | 1.18(1.11-1.24) |
| **Follow-up begin at birth** | unexposed | 2113406 | 78688 | 2.06 | 1.0(ref) | 1.0(ref) |
| **(N=2,137,436)** | exposed | 24030 | 1343 | 3.78 | 1.20(1.13-1.26) | 1.17(1.11-1.23) |
| **Follow-up begin at half one year** | unexposed | 2092958 | 77171 | 2.05 | 1.0(ref) | 1.0(ref) |
| **(N=2,116,280)** | exposed | 23322 | 1307 | 3.83 | 1.20(1.13-1.26) | 1.17(1.11-1.24) |
| **Follow-up begin at 3 years old** | unexposed | 2000587 | 75412 | 2.18 | 1.0(ref) | 1.0(ref) |
| **(N=2,020,419)** | exposed | 19832 | 1118 | 4.21 | 1.20(1.13-1.28) | 1.17(1.11-1.24) |
| **Follow-up begin at 5 years old** | unexposed | 1907034 | 73656 | 2.33 | 1.0(ref) | 1.0(ref) |
| **(N=1,924,008)** | exposed | 16974 | 962 | 4.68 | 1.23(1.15-1.31) | 1.19(1.12-1.27) |
| **Restricted to children born after 1980** | unexposed | 1992475 | 66165 | 1.96 | 1.0(ref) | 1.0(ref) |
| **(N=2,012,998)** | exposed | 20523 | 1048 | 3.65 | 1.22(1.14-1.29) | 1.19(1.12-1.26) |
| **Restricted to children born after 1991** | unexposed | 1469569 | 26097 | 1.41 | 1.0(ref) | 1.0(ref) |
| **(N=1,478,837)** | exposed | 9268 | 231 | 2.37 | 1.26(1.10-1.43) | 1.23(1.08-1.40) |
| **Restricted to children born after 1994** | unexposed | 1294787 | 18399 | 1.27 | 1.0(ref) | 1.0(ref) |
| **(N=1,301,636)** | exposed | 6849 | 119 | 1.85 | 1.14(0.95-1.37) | 1.13(0.94-1.36) |
| **Complete case analysis** | unexposed | 1362712 | 24163 | 1.41 | 1.0(ref) | 1.0(ref) |
| **(N=1,371,006)** | exposed | 8294 | 207 | 2.38 | 1.26(1.10-1.44) | 1.23(1.07-1.41) |
| **Spline function for birth weight, Apgar score, maternal age and calendar year** | unexposed | 2075691 | 76862 | 2.08 | 1.0(ref) | 1.0(ref) |
| **(N=2,098,659)** | exposed | 22968 | 1286 | 3.86 | 1.19(1.13-1.26) | 1.17(1.11-1.24) |

Abbreviations: CVD, cardiovascular disease; cHR, crude hazard ratio; aHR, adjusted hazard ratio.

[a] Adjusted for preterm birth, birth weight, singleton birth, parity, Apgar score at 5 minutes, maternal age, maternal education, maternal smoking during pregnancy, sex of child, and calendar period, maternal hypertensive disorder of pregnancy, parental history of CVD before child birth, parental history of diabetes before child birth.

© 2024 Huang C et al. *JAMA Network Open.*

**eFigure 1. Flowchart of the Study Participants**

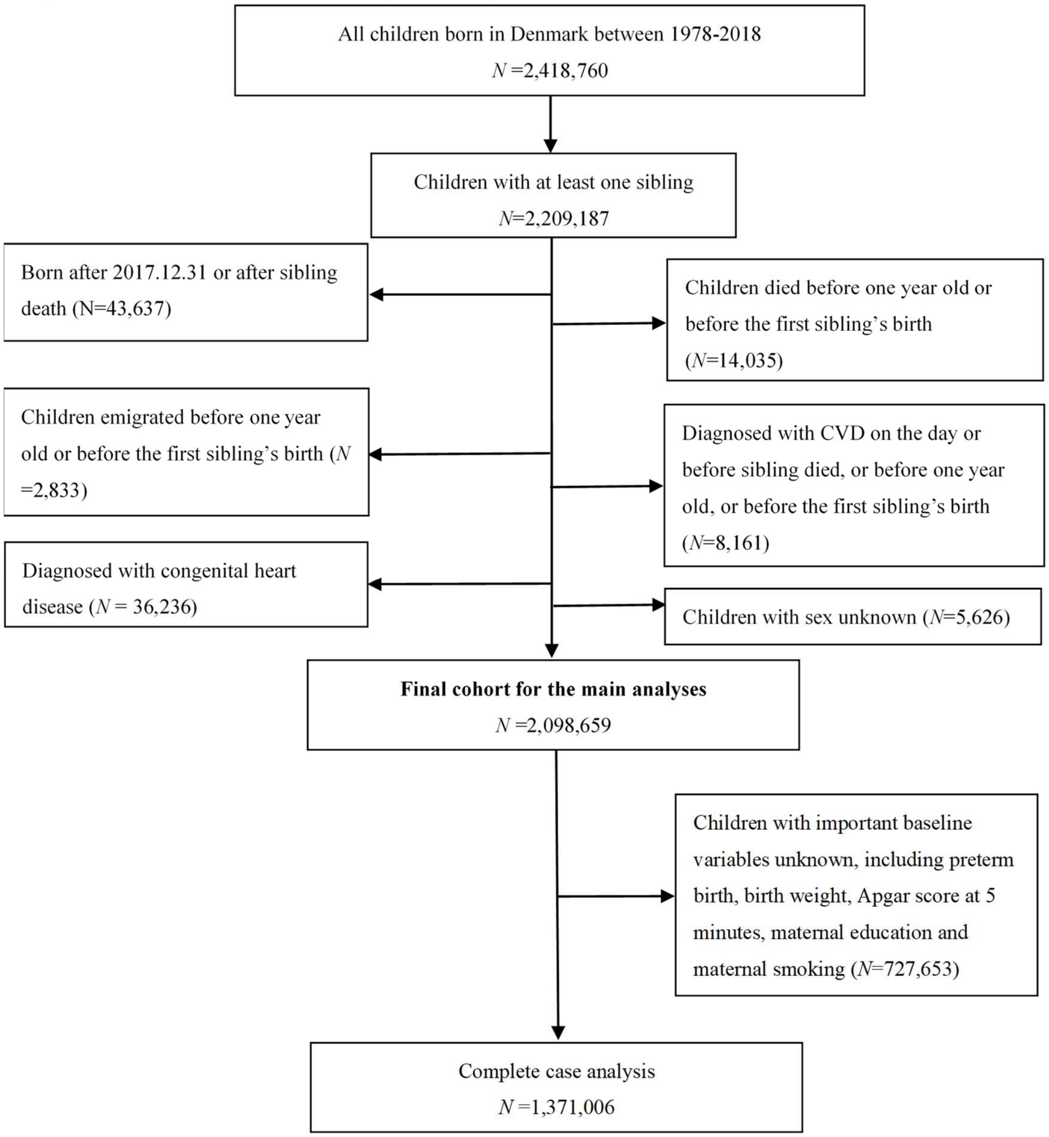


Abbreviation: CVD, cardiovascular disease.

© 2024 Huang C et al. *JAMA Network Open*.

**eFigure 2. Directed Acyclic Graph (DAG) Used to Select Covariates for Confounding Control[a b]**

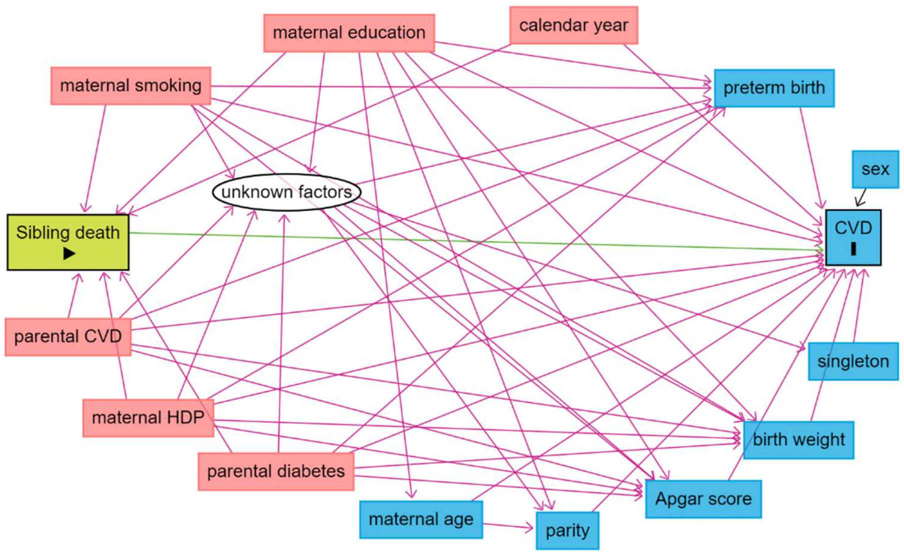


[a] HDP, hypertensive disorder of pregnancy; CVD, cardiovascular disease; sex, sex of child; calendar year refers to birth year of individual.

[b] We assume that maternal smoking may influence sibling death, CVD, preterm birth, singleton, birth weight, Apgar score and unknown factors; maternal education may influence sibling death, CVD, preterm birth, singleton, birth weight, Apgar score, parity, maternal age and unknown factors; calendar year may influence sibling death and CVD; parental CVD may influence sibling death, CVD, preterm birth, birth weight, Apgar score and unknown factors; parental diabetes may influence sibling death, CVD, preterm birth, birth weight, Apgar score and unknown factors; maternal HDP may influence sibling death, CVD, preterm birth, birth weight, Apgar score and unknown factors; maternal age may influence CVD and parity; Apgar score, birth weight, preterm birth, parity, singleton and sex may influence CVD; Apgar score, birth weight, preterm birth, parity, singleton may be influenced by unknown factors.

© 2024 Huang C et al. *JAMA Network Open*.

**eFigure 3. The Log-Minus-Log Survival Curve for Sibling Death and Overall CVD**

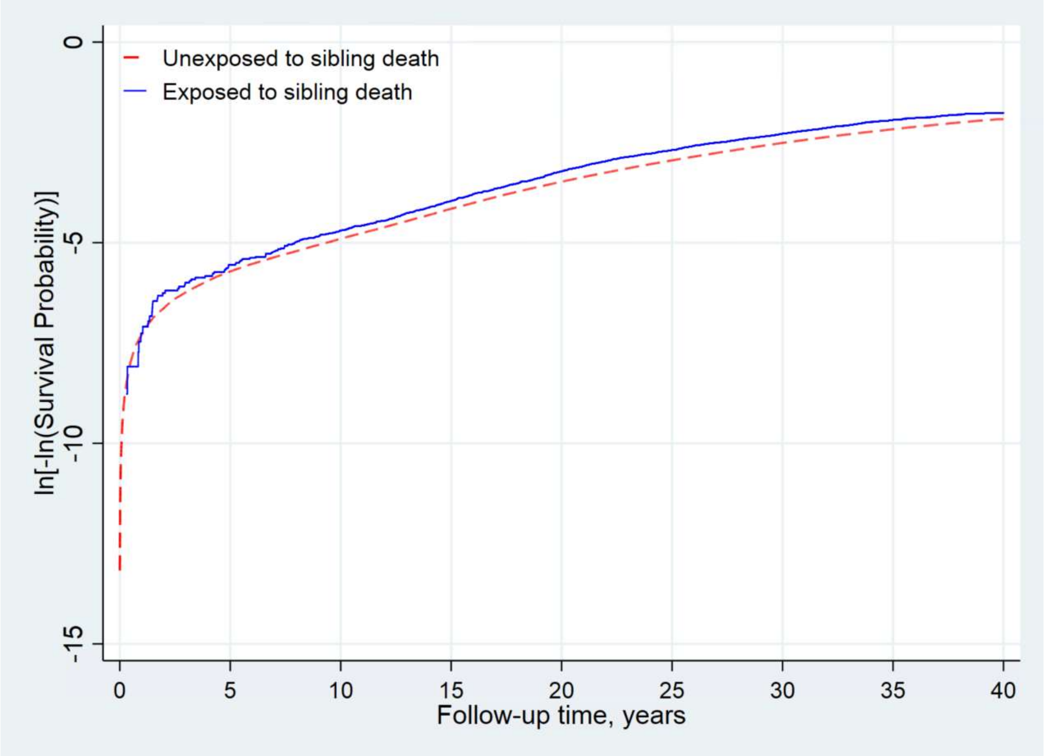


© 2024 Huang C et al. *JAMA Network Open.*
