# Supplementary material for: Sibling Death in Childhood and Early Adulthood and Risk of Early-Onset Cardiovascular Disease
Source: JAMA Netw Open. 2024 Jan 8;7(1):e2350814. doi: 10.1001/jamanetworkopen.2023.50814 (PMC10774991; doi:10.1001/jamanetworkopen.2023.50814)
Supplement: Supplement 2. — Data Sharing Statement [file jamanetwopen-e2350814-s002.pdf]

Data Sharing Statement

Huang. Sibling Death in Childhood and Early Adulthood and Risk of Early-Onset Cardiovascular Disease. *JAMA Netw Open*. Published January 08, 2024. doi:10.1001/jamanetworkopen.2023.50814

Data
Data available: No

Additional Information
Explanation for why data not available: All data is stored at the secure platform of Denmark Statistics, which is the central authority on Danish statistics with the mission to collect, compile and publish statistics on the Danish society. Due to restrictions related to Danish law and protecting patient privacy, the combined set of data as used in this study can only be made available through a trusted third party, Statistics Denmark (https://www.dst.dk/en/kontakt).
